# Electron Beam Structuring of Ti6Al4V: New Insights on the Metal Surface Properties Influencing the Bacterial Adhesion

**DOI:** 10.3390/ma13020409

**Published:** 2020-01-15

**Authors:** Sara Ferraris, Fernando Warchomicka, Fatemeh Iranshahi, Lia Rimondini, Andrea Cochis, Silvia Spriano

**Affiliations:** 1 Department of Applied Science and Technology, Politecnico di Torino, 10129 Torino, Italy; sara.ferraris@polito.it; 2 Institute of Materials Science, Joining and Forming, Graz University of Technology, A-8010 GRAZ, Austria; fernando.warchomicka@tugraz.at (F.W.); fatemeh.iranshahi@tugraz.at (F.I.); 3 Department of Health Sciences, Università del Piemonte Orientale, 28100 Novara, Italy; lia.rimondini@med.uniupo.it (L.R.); andrea.cochis@med.uniupo.it (A.C.); 4 Center for Translational Research on Autoimmune & Allergic Diseases—CAAD, 28100 Novara, Italy

**Keywords:** Titanium, bacteria adhesion, microstructure, electron beam structuring, fibroblast alignment

## Abstract

Soft tissue adhesion and infection prevention are currently challenging for dental transmucosal or percutaneous orthopedic implants. It has previously been shown that aligned micro-grooves obtained by Electron Beam (EB) can drive fibroblast alignment for improved soft tissue adhesion. In this work, evidence is presented that the same technique can also be effective for a reduction of the infection risk. Grooves 10–30 µm wide and around 0.2 µm deep were obtained on Ti6Al4V by EB. EB treatment changes the crystalline structure and microstructure in a surface layer that is thicker than the groove depth. Unexpectedly, a significant bacterial reduction was observed. The surfaces were characterized by field emission scanning electron microscopy, X-ray diffraction, confocal microscopy, contact profilometry, wettability and bacterial adhesion tests. The influence of surface topography, microstructure and crystallography on bacterial adhesion was systematically investigated: it was evidenced that the bacterial reduction after EB surface treatment is not correlated with the grooves, but with the microstructure induced by the EB treatment, with a significant bacterial reduction when the surface microstructure has a high density of grain boundaries. This correlation between microstructure and bacterial adhesion was reported for the first time for Ti alloys.

## 1. Introduction

The main challenges of titanium surfaces facing soft tissues are formation of a well anchored and oriented soft tissue, as well as the reduction of bacterial contamination [1].

It is widely known that surface micro/nano patterns can affect cellular and bacterial adhesion [2] and that different types of cells have different responses to surface topographies. In particular, fibroblasts are rugo-phobic (their adhesion is reduced on rough surfaces, e.g., 300 nm height holes/pillars [3]), but they can be oriented by aligned grooves, following the contact guidance phenomenon [4]. On the other hand, bacteria are rugo-philic, and an increase in bacterial adhesion with surface roughness has been widely reported [2,5]; an average surface roughness of 0.2 µm has been indicated as the threshold below which bacterial adhesion does not increase [4,6,7], and 150 µm has been indicated as the upper limit above which no more variation in bacterial attachment can be observed [8]. Fibroblast cells and bacteria are also different with respect to their dimensions (the former being 15–20 µm and the latter being 1–2 µm) and their ability to follow surface patterns (bacteria are rigid, while cells are able to deform in order to develop focal contacts in surface adhesion phenomena), so they can be differently stimulated by material surface features [2]. Following this rationale, the authors defined the optimal ranges of dimensions for grooves (W = width W > 100 nm and W < 70 µm; D = depth, D > 35 nm, average roughness (Ra) < 0.2 µm) able to stimulate fibroblast orientation without increasing bacterial adhesion [9]. Surfaces with topographies accordingly designed were produced on commercially pure titanium with grooves obtained by mechanical polishing [10] or electron beam (EB) structuring [11]. An effective fibroblast alignment was observed on these surfaces [6]. Unexpectedly, initial evidence of reduced bacterial adhesion on the EB structured surfaces was also observed [11]. Since EB-structuring induces, in addition to topographical modifications, alterations in surface crystallography and microstructure, a possible correlation of these features with the bacterial adhesion was hypothesized. This phenomenon is poorly explored in the literature; a possible influence of the grain dimension and grain boundaries has been reported for stainless steel [12], and an effect of martensite crystallographic structure and associated peculiar nanotopography have been mentioned for titanium [13], but few research works have focused on this topic.

The aim of this research is the in-depth investigation of the effect of topography, crystallography and microstructure on bacterial adhesion for EB structured Ti6Al4V surfaces presenting micro-grooves aimed at fibroblast alignment. For the first time, a correlation between surface microstructure and bacterial adhesion has been demonstrated for Ti6Al4V.

## 2. Materials and Methods

The Ti6Al4V samples were mirror polished (MP) with SiC abrasive papers and colloidal silica suspension and finally washed (acetone and distilled water in ultrasonic bath).

Surface structuring was performed by an Electron Beam machine (Probeam EBG 45-150 K14, GmbH & Co. KGaA, Gilching, Germany) as described in [11], obtaining 10 and 30 µm wide grooves (EB10 and EB30, Table 1).

Some EB10 samples underwent a thermal treatment at 1030 °C (5 min, 10^−4^ mbar, 100 K/min cooling rate) in a dilatometer (Bähr-Thermoanalyse DIL805 A/D, Bähr Thermoanalyse GmbH, Hüllhorst, Germany) in high vacuum (< 10^−5^ mbar) with the aim of removing the residual stresses and to form a fine α lamellae microstructure (EB10HT). Additionally, samples with the heat treatment previously described were immersed during 4 min in HF 1% solution to reveal the microstructural features (EB10HT etch—Table 1).

Some EB structured samples were polished with a specific protocol; a surface layer thick enough to remove the grooves was abraded, but the microstructure induced by EB was kept because it is developed in a thicker layer (P10 and P30—Table 1).

Surface appearance and microstructure were investigated by Field Emission Scanning Electron Microscopy (FESEM—TESCAN Mira 3-15kV-beam size 5 nm, Tescan, Brno, Czech Republic). Surface morphology was analyzed qualitatively with secondary electron (SE) detector, using a tilt angle of 60° for a better observation of the grooves and the microstructural features at the surface of the material. The microstructure of the structured area and the base material were observed in backscattered-electron mode (BSE) detector to reveal the presence of α, β and α’martensite phases.

Moreover, surface topography was analyzed with a confocal microscope (LSM 900, Zeiss, Oberkochen, Germany; 50x/0.95 objective) and surface roughness (Ra) measured with a contact profiler (Intra Touch, Taylor Hobson, Leicester, United Kingdom). The average and maximum grooves’ depth was measured on the topography profiles of three 250x250 micron areas.

Crystalline structure was investigated by X-Ray Diffraction (XRD, PANalytical X’Pert Pro PW 3040160 Philips, Malvern Panalytical, Egham, United Kingdom) and the spectra were analyzed by XPERT High Score software (2.2b).

Surface wettability was investigated by means of contact angle measurements (sessile drop method, DSA-100, KRÜSS GmbH, Hamburg, Germany) using ultrapure water as wetting fluid.

For biological assays, specimens were realized as 1 cm-side squares, heat sterilized (180 °C, 1 h) and stored at room temperature until use and gently located into the wells of a 12-multiwell plate. Cell groove-driven orientation was assayed using primary human gingival fibroblasts (HGF, ATCC PCS-201-018, American Type Culture Collection, Manassas, VA, USA). Cells were cultivated with Minimal Essential Medium alpha-modification (α-MEM, from Sigma, Milan, Italy) supplemented with 10% fetal bovine serum and 1% antibiotics at 37 °C, in a 5% CO_2_ atmosphere. Once they reached 70–80% confluence, cells were detached by trypsin-EDTA solution (from Sigma, Milan, Italy), counted by Bürker chamber and seeded directly onto specimen’s surface at a final density of 5 × 10^3^ cells/specimen. The plate was incubated at 37 °C, 5% CO_2_ for 48 h to allow complete cell adhesion and spread; then, cell orientation was investigated by means of f-actins cytoskeleton filaments visualization by phalloidin. Briefly, cells were fixed with 4% formaldehyde (in PBS) for 5 min at room temperature, permeabilized 10 min with 0.1% Triton (in PBS), and finally stained 45 min with phalloidin (1:500 in PBS, from AbCam, UK). Then, cells were washed with PBS and co-stained with 4′,6-diamidino-2-phenylindole (DAPI, from Sigma, Milan, Italy) to visualize nuclei. Images were collected by fluorescent microscope (Leica DM 6500, Leica Systems, Basel, Switzerland).

Bacterial adhesion was evaluated with a strong biofilm former multi-drug resistant certified *Staphylococcus aureus* strain (SA, ATCC 43300, American Type Culture Collection, Manassas, VA, USA). Bacteria were cultivated in Luria Bertani broth (LB, Sigma, Milan, Italy) at 37 °C overnight; then, bacteria were diluted 1:10 by mixing 1 mL of the broth culture with 9 mL of fresh LB medium and incubated again at 37 °C for 3 h to achieve the logarithmic growth phase. Lastly, bacteria were diluted in fresh medium until the solution optical density (o.d.) was 0.001 at 600 nm corresponding to a final concentration of 1 × 10^5^ cells per mL. The o.d. was evaluated by spectrophotometer (Spark, Tecan, Basel, Switzerland) using fresh medium as blank.

To study specimens’ antibacterial properties, 1 mL of the 1 × 10^5^ bacteria suspension was used to submerge the samples that were previously located into the wells of a 12-multiwell plate. The plate was incubated for 90 min at 37 °C under agitation (120 rpm) to force the separation between adherent biofilm bacteria and floating planktonic ones (separation phase). Afterwards, the supernatants containing planktonic bacteria were removed and replaced with fresh LB medium to cultivate adhered biofilm (separation phase) for 24–48–72 h [14,15]. At each time point, adhered biofilm bacteria metabolism was evaluated by the colorimetric alamar blue assay (alamarBlue^®^, Life Technologies, Milan, Italy) [12,13]; briefly, the supernatants were removed from the well and replaced by the ready-to-use alamar solution. The plate was incubated for 3 h at 37 °C in the dark and then 100 mL were collected from each sample and moved to a new black-bottom 96-multiwell plate. The fluorescence signal was evaluated by spectrophotometer (Spark, Tecan, Basel, Switzerland) using a 590 nm wavelength. Results were expressed as relative fluorescence units (RFU) and alamar solution was used as blank.

After 72 h, the number of adhered biofilm bacteria was further evaluated by the colony forming units (CFU) count. Briefly, specimens were washed 3 timed with PBS to remove non-adherent bacteria and then vortexed and sonicated 3 times (30 s each) in 1 mL of PBS to detach biofilm. Then, aliquots of 100 μL were collected and used to perform six 10-fold serial dilutions; finally, the number of CFU was calculated using Equation (1) [15]:

*CFU = [(number of colonies x dilution factor)^∧(serial dilution)^]* (1)

where:

*number of colonies* = countable single round colonies;
*dilution factor* = dilution made from the initial 1mL suspension;
*serial dilution* = 1–6 10-fold dilution areas where colonies were counted.

All experiments were performed in triplicate. Data were analyzed using SPSS software (v25, IBM, NY, USA) by one-way ANOVA followed by the Tukey’s test as post hoc analysis. The significance level was set at p < 0.05.

## 3. Results

Firstly, the sample surface appearance and microstructure were observed by FESEM observation on a case by case basis. The crystallographic structure of the grains was as first deduced from FESEM observation according to well-known metallography of Ti6Al4V and then confirmed by XRD.

**Figure 1:**
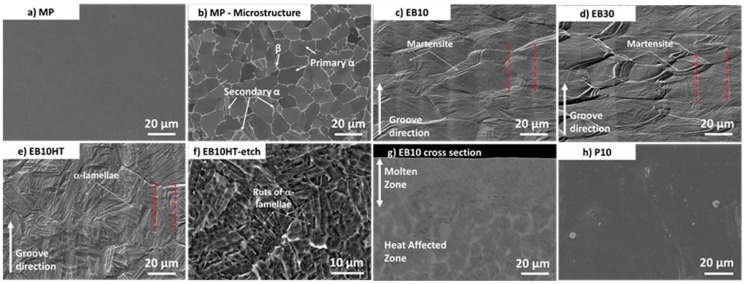
FESEM images of the samples: (**a**) MP sample (Secondary Electron (SE) image); (**b**) MP sample (BSE image); (**c**) EB 10 sample (SE image); (**d**) EB 30 sample (SE image); (**e**) EB10HT sample (SE image); (**f**) EB10HT-etch sample (SE image); (**g**) cross section of the EB10 sample (SE image); (**h**) P10 sample (SE image).

At FESEM observation, MP samples show smooth appearance (Figure 1a) and the bimodal microstructure of wrought Ti6Al4V alloy (equiaxed primary α-grains, secondary α lamellae and β-phase along grain boundaries, visible by backscattered-electron mode (BSE) detector).

10 and 30 µm wide grooves are visible on EB10 and EB30 according to the EB surface structuring process (as an example, the groove valleys are highlighted by red dotted lines in Figure 1c–e); these samples have a typical martensitic microstructure. The cross section of the EB structured samples evidence a martensitic zone provoked by fast cooling of the molten pool during EB structuring and a wide heat affected zone with a mixture of α’, primary α and β-phase. The molten zone is about 40 µm thick, and the heat-affected zone is estimated to be 100 µm. It can be concluded that the thermal modified surface layer is quite thicker than the grooves depth (see below).

The thermal treatment (EB10HT) does not alter the grooves morphology (as an example the grooves edges are highlighted by red dotted lines), but it induces the formation of α lamellae colonies. After etching (EB10HT-etch), the surface becomes more complex and slightly more rough due to selective etching of the phases; ruts can be observed where α lamellae were present before etching, due to the complexity of the surface texture, the grooves are hardly observable on these surfaces (even if they are still present).

EB structured and polished samples (P10) have a smooth appearance, and grooves are no longer visible, as expected; the depth of the abraded material during polishing was checked to be lower than the heat affected zone due to EB structuring.

**Figure 2:**
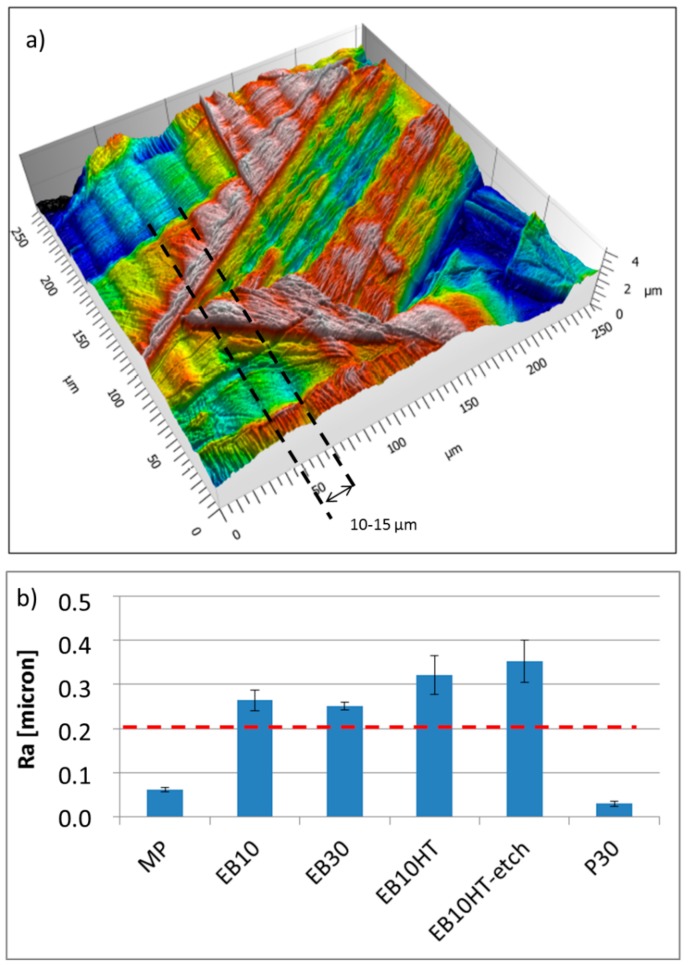
(**a**) Confocal microscopy 3D image of EB10 sample, (**b**) roughness measurements (contact profiler).

Groove presence on the EB-structured samples is confirmed by confocal microscopy observations (an example for EB10 sample is reported in Figure 2a, where an example of groove is evidenced by dotted lines). The same picture highlights also the presence of martensite inducing high density of grain boundaries and lamellae very close each other, protruding on a nanometric scale. The average and maximum groove depths are 270 and 310 nm, respectively, as measured from the topography profiles acquired on the surface.

Roughness measurements evidence, as expected, a nanometric scale roughness on the mirror polished samples (MP) and on the samples polished after EB structuring (P30 reported as example), confirming their smooth appearance observed at FESEM and confirming also the ability of the developed polishing protocol to completely remove the grooves. On the other hand, a roughness (Ra) close to 0.2 µm was measured on the EB structured samples (slightly higher after etching, in accordance with the FESEM observations) confirming the grooves presence and surface topography changes connected to the EB treatment.

**Figure 3:**
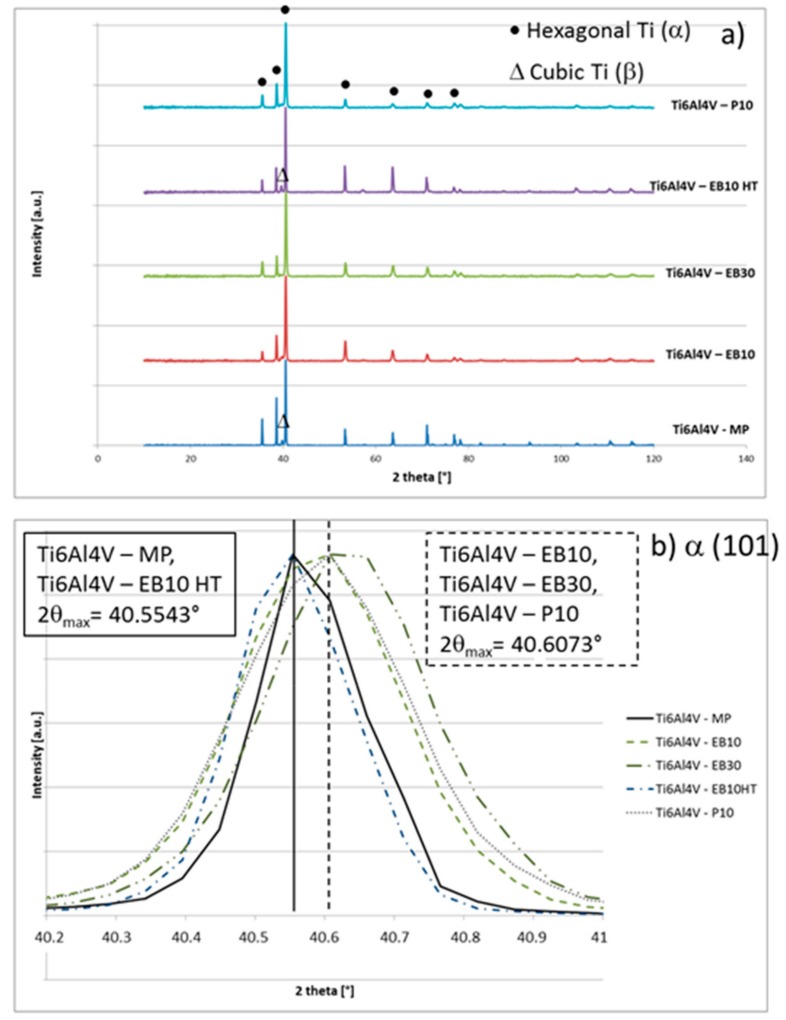
XRD spectra (normalized to the highest peak) (**a**) whole spectra, (**b**) detail of the highest peak.

XRD spectra of the samples are reported in Figure 3. All the samples show a prevalence of hexagonal Ti (α phase) and a small signal of cubic Ti (β phase), which is more evident on MP and EB10HT. A shift of the main α peak (101) toward slightly higher values can be evidenced for the EB structured samples (from 40.554° to 40.607°) and attributed to the presence of α’ martensite, as suggested by [16] and previously observed on EB structured Ti-cp [11]. The presence of martensite on EB10 and EB30 is in agreement with the FESEM observations discussed above. The shift persists after polishing (P10), confirming that the process removed the grooves, but not all the thermal modified layer induced by EB-structuring. On the other hand, the shift is completely recovered on EB10HT according to the expected removal of martensite by the thermal treatment.

The topographical, crystallographic structural and microstructural characteristics of the samples are reported and summarized in Table 2.

The results of the contact angle measurements are reported in Table 3.

All values are comprised in the 70–80° range, which is in line with the contact angle values for the titanium surfaces reported in the literature [17]. EB treatment slightly increases the contact angle values in accordance with an increase in surface roughness; however, variations are so limited that no effect on bacterial adhesion can be reasonably attributed to surface wettability.

**Figure 4:**
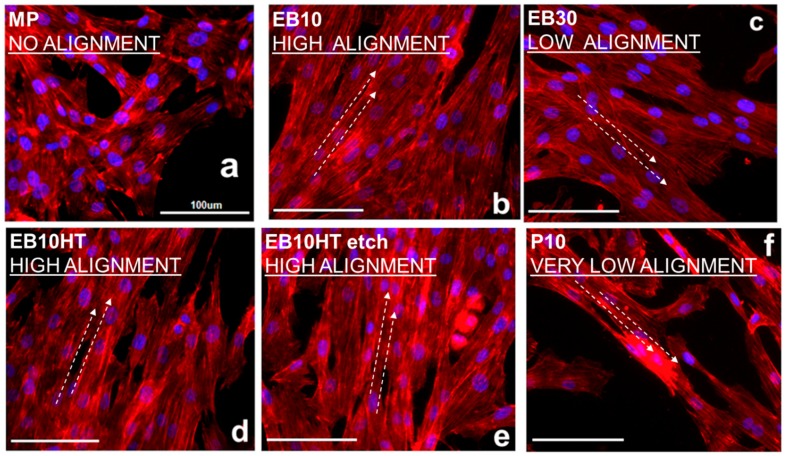
Fibroblast alignment on the different samples after 48 h culture: (**a**) MP sample; (**b**) EB10 sample; (**c**) EB30 sample; (**d**) EB10HT sample; (**e**) EB10HT etch sample; (**f**) P10 sample.

Fibroblasts alignment on the different surfaces is shown in Figure 4. As previously shown by the authors onto commercially pure titanium (Ti-cp) [11], primary fibroblasts successfully spread along the EB grooves’ topography, thus showing an oriented alignment, which is better defined onto 10 µm sized grooves (Figure 4b). 30-µm-sized grooves (Figure 4c) resulted in the alignment of fibroblast cells which having a characteristic dimension of 15–25 µm, according to previous works on Ti-cp [11]. Alignment is maintained after thermal treatment (EB10HT, Figure 4d) and etching (EB10HTetch, Figure 4e), consistently with groove maintenance. On the other hand, no alignment is observed on MP (Figure 4a) and P10 (Figure 4f) samples according to groove absence and grooves removal by polishing, respectively.

**Figure 5:**
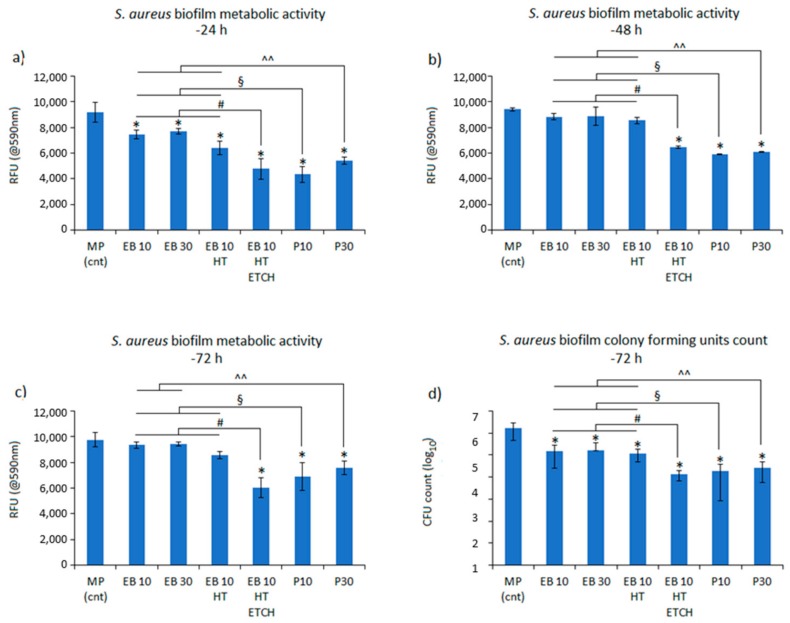
Bacterial metabolic activity evaluation (**a** = 24 h, **b** = 48 h, **c** = 72 h) and (**d**) number count of the viable colonies after 72 h. The bars represent means and standard deviations.

The bacterial adhesion (Figure 5) did not increase after EB structuring with respect to a control polished sample (MP), as expected considering that roughness of EB structured samples is not far from 0.2 µm (the threshold reported in the literature as the limit to not increase bacterial adhesion) [11].

Beyond the expectations, a significant reduction of the bacteria metabolism was noticed for all the EB structured samples (EB10, EB30, EB10HT, EB10HT-etch, P10, P30) in comparison with the control (MP) after 24 h (Figure 5a, p < 0.05, indicated by *). Conversely, after 48 (Figure 5b) and 72 h (Figure 5c) only the specimens EB10HT-etch, P10 and P30 resulted as significant in comparison with smooth MP control (p < 0.05, indicated by *).

Widening the comparison among the EB structured samples, further significant differences were noticed as soon as the microstructure was clearly exposed by etching and polishing steps: in fact, EB10HT-etch and P10 and P30 had a significantly lower bacteria metabolism in comparison with EB10 and EB30 at each time points. Specifically, after 24 h (Figure 5a), EB10HT-etch was significant towards EB10, EB30 and EB10HT (p < 0.05, indicated by #), P10 was significant towards EB10, EB30 and EB10HT (p < 0.05, indicated by §) and P30 was significant towards EB10, EB30 and EB10HT (p < 0.05, indicated by ^^). After 48 h (Figure 5b), the same significant differences as 24 h were detected while after 72 h (Figure 5c), EB10HT-etch was significant towards EB10, EB30 and EB10HT (p < 0.05, indicated by #), P10 was significant towards EB10, EB30 and EB10HT (p < 0.05, indicated by §) and P30 was significant towards EB10 and EB30 (p < 0.05, indicated by ^^).

In agreement with the previous results related to bacteria metabolism, the CFU count (Figure 5d) after 72 h showed that a lower number of viable colonies adhered to the EB structured surfaces in comparison to the MP control ones (p < 0.05, indicated by the *). Moreover, when the comparison was extended to the EB structured samples, a similar trend with the above described alamar blue assay was noticed; in fact, EB10HT-etch was significant towards EB10, EB30 and EB10HT (p < 0.05, indicated by #), P10 was significant towards EB10, EB30 and EB10HT (p < 0.05, indicated by §) and P30 was significant towards EB10, EB30 and EB10HT (p < 0.05, indicated by ^^).

## 4. Discussion

Considering the promising contact guidance effect of EB grooves on fibroblast alignment and the relevant issue of infection risk in dental and orthopedic implants, the present work aims at investigating the eventual relationship between bacteria adhesion and surface features of EB structured Ti6Al4V alloy with a particular focus on the possible effect related to the metallic microstructure/crystalline structure. In particular, the stated hypothesis deals with the evidence that when compared to the eukaryotic cells, the bacteria hold a less elastic membrane and a higher stability of the shape maintenance: as a consequence, they result much more sensitive to surface topography because of strong limitation for adhesion as restricting availability of berth points [18] as it was previously demonstrated by the authors for different implantable metallic devices [19]. Therefore, to further study the effect towards cells and bacteria of the microstructure/crystalline structure, here different EB-treated surfaces were assayed with or without etching and polishing to understand whether the exposure of the metallic microstructure/crystalline structure can be a factor influencing the biological response. In fact, the results demonstrated that the test specimens belong the same surface chemistry (all the surfaces show a layer of native titanium oxide) as well as surface wettability thus ascribing all the observed differences to the microtopography, microstructure and/or crystalline structure.

Summarizing the results of the topographical, structural and microstructural analyses of the explored samples, EB10 and EB30 show grooves, martensite and microstructure with high density of grain boundaries (due to martensite); EB10HT shows grooves and a high density of grain boundaries (due to α-lamellae), but no martensite; P10 and P30 show martensite and a high density of grain boundaries (due to martensite and α-lamellae), but no grooves; EB10HT-etch show high density of grain boundaries (due to α-lamellae), no grooves and no martensite.

The influence towards cells orientation showed by the EB samples grooves is an expected result as it is well known that fibroblasts are sensitive to surface topography by adapting cytoskeleton spread [20]. Here, we basically confirmed this evidenced in relation to grooved alloy surfaces obtained by new techniques, such as EB.

The results obtained by antibacterial evaluation are worthy of more attention. In fact, despite an increase of their roughness, the surfaces with grooves created through the EB treatment showed a reduction of bacterial adhesion compared to the mirror polished ones (MP). Moreover, the MP and P10/P30 samples have a similar surface texture (smooth appearance) and average roughness (Ra around tens of nanometers), but bacterial adhesion is significantly reduced on the P10/30 surfaces compared to the MP ones: this shows that the EB treatment is able to reduce the bacterial adhesion even if the grooves are removed by polishing and roughness (in terms of Ra) is almost the same. The P10/30 samples are characterized by the microstructure/crystalline structure due to EB treatment with high density of grain boundaries. Accordingly, here we evidenced that a common characteristic for the surfaces which showed reduced bacterial adhesion is the high density of grain boundaries, that can be supposed to be the determinant feature affecting bacteria adhesion and metabolism on the explored samples. As confirmation, if the microstructure with high density of grain boundaries of Ti6Al4V alloy is evidenced by etching, as on EB10HT-etch, the effect is much more visible. According to the here presented results, the evidence of the effect of surface microstructure of titanium alloys on bacterial adhesion was here presented for the first time.

## 5. Conclusions

Oriented grooves were obtained on Ti6Al4V by EB-structuring, which also induces alteration of surface crystallographic structure and microstructure. Grooves with ten µm width showed the ability to align fibroblast cells; moreover, a statistically significant reduction of bacterial adhesion was observed on the EB structured samples, in absence of antibacterial agents and even after removal of the grooves by polishing: it can be ascribed to the presence of a microstructure with high density of grain boundaries after EB surface treatment. The relationship between the microstructure of titanium alloys and anti-adhesive activity against bacteria is here presented for the first time and it opens a new strategy for the development of innovative antiadhesive/antibacterial metallic materials.

## Figures and Tables

**Table 1:** Ti6Al4V sample names and processing.

Sample Name	Processing
MP	Mirror polishing
EB10	Mirror polishing + EB-structuring (EB10)
EB30	Mirror polishing + EB-structuring (EB30)
EB10HT	Mirror polishing + EB-structuring (EB10) + heat treatment
EB10HT-etch	Mirror polishing + EB-structuring (EB10) + heat treatment + etching
P10	Mirror polishing + EB-structuring (EB10) + mirror polishing
P30	Mirror polishing + EB-structuring (EB30) + mirror polishing

**Table 2:** Ti6Al4V sample topography, structure and microstructure.

Sample Name	Surface Appearance	Ra [µm]	Crystallographic Structure	Microstructure
MP	Smooth	0.06	Prevalence of hexagonal Ti (α)	Bimodal (equiaxed primary α grains, secondary α lamellae and β phase)
EB10	10 µm grooves	0.26	Prevalence of hexagonal Ti martensite (α’)	Martensitic microstructure
EB30	30 µm grooves	0.25	Prevalence of hexagonal Ti martensite (α’)	Martensitic microstructure
EB10HT	10 µm grooves	0.32	Prevalence of hexagonal Ti (α)	α lamellae colonies
EB10HT-etch	Etched	0.35	Prevalence of hexagonal Ti (α)	α lamellae colonies
P10	Smooth		Prevalence of hexagonal Ti martensite (α’)	Martensitic microstructure
P30	Smooth	0.03	Prevalence of hexagonal Ti martensite (α’)	Martensitic microstructure

**Table 3:** Contact angle measurements.

Sample	Cotact Angle [°]
MP	74 ± 4
EB10	81 ± 4
EB30	81 ± 4
EB10HT	68 ± 3
EB10HTetch	80 ± 3
P10	75 ± 2
